# Night-Restricted Feeding Improves Gut Health by Synchronizing Microbe-Driven Serotonin Rhythm and Eating Activity-Driven Body Temperature Oscillations in Growing Rabbits

**DOI:** 10.3389/fcimb.2021.771088

**Published:** 2021-12-17

**Authors:** Qiang-Jun Wang, Yao Guo, Ke-Hao Zhang, Lei Zhang, Shi-Xia Geng, Chun-Hua Shan, Peng Liu, Meng-Qi Zhu, Qiong-Yu Jin, Zhong-Ying Liu, Mei-Zhi Wang, Ming-Yong Li, Man Liu, Lei An, Jian-Hui Tian, Zhong-Hong Wu

**Affiliations:** ^1^ State Key Laboratory of Animal Nutrition, College of Animal Science and Technology, China Agricultural University, Beijing, China; ^2^ National Rabbit Industry Technology System Qingdao Comprehensive Experimental Station, Qingdao, China; ^3^ Key Laboratory of Animal Genetics, Breeding and Reproduction of the Ministry of Agriculture and Rural Affairs, National Engineering Laboratory for Animal Breeding, College of Animal Science and Technology, China Agricultural University, Beijing, China

**Keywords:** night-restricted feeding, diurnal rhythm, gut microbiota, serotonin, body temperature

## Abstract

The circadian misalignment of the gut microbiota caused by unusual eating times in adult animals is related to disease development. However, whether the composition and diurnal rhythm of gut microbiota can be optimized by synchronizing the window period of eating with natural eating habits to reduce the risk of diarrhea remains unclear, especially in growing animals. In this study, 108 5-week-old weaned rabbits (nocturnal animals) were randomly subjected to daytime feeding (DF) and night-restricted feeding (NRF). At age 12 weeks, six rabbits were selected from each group, and caecum and cecal contents, as well as serum samples were collected at 4-h intervals during 24 h. Overall, NRF was found to reduce the risk of diarrhea in growing rabbits, improved the diurnal rhythm and abundance of beneficial microorganisms, along with the production of beneficial metabolites, whereas reduced the abundance of potential pathogens (*Synergistes*, *Desulfovibrio*, and *Alistipes*). Moreover, NRF improved diurnal rhythm of tryptophan hydroxylase isoform 1 and serotonin. Furthermore, NRF strengthened the diurnal amplitude of body core temperature, and promoted the diurnal expression of intestinal clock genes (*BMAL1*, *CLOCK*, *REV-ERBα*, and *PER1*), and genes related to the regulation of the intestinal barrier (*CLAUDIN-1*), and intestinal epithelial cell self-proliferation and renewal (*BMI1*). *In vitro* simulation experiments further revealed that synchronization of microbial-driven serotonin rhythm and eating activity-driven body temperature oscillations, which are important zeitgebers, could promote the diurnal expression of clock genes and *CLAUDIN-1* in rabbit intestinal epithelial cells (RIEC), and enhance RIEC proliferation. This is the first study to reveal that NRF reprograms the diurnal rhythm of the gut microbiome, promotes the diurnal expression of clock genes and tight junction genes *via* synchronization of microbial-driven serotonin rhythm and eating activity-driven body temperature oscillations, thereby improving intestinal health and reducing the risk of diarrhea in growing rabbits. Collectively, these results provide a new perspective for the healthy feeding and management of growing animals.

## Introduction

Unhealthy adult lifestyles that include shift work, nighttime social activities and jet lag are becoming more prevalent, and consequently, associated health problems have also become more prominent (Parsons et al., 2015; Koshy et al., 2019). Epidemiological investigations have determined that unhealthy lifestyle-promoted metabolic syndromes and intestinal inflammatory diseases result in heavy global economic burden (Bishehsari et al., 2020). However, the increasing academic burden on students has become a serious concern, as it has contributed toward the annual increase in insufficient sleep and nocturnal diets in children, which in turn are associated to higher rates of obesity, cardiovascular disease, and chronic intestinal inflammatory diseases during childhood (Eastman et al., 2015). Therefore, in recent years, unhealthy lifestyles causing disturbances in the diurnal rhythm of intestinal microorganisms have become the focus of research (Asher and Sassone-Corsi, 2015; Murakami and Tognini, 2019; Bishehsari et al., 2020). However, there are still many challenged associated with studying the diurnal rhythms of human gut microbes, such as continuous multi-temporal sampling, differences in environmental and dietary composition, and particularly the lack of studies on children and growing animals (Frazier and Chang, 2020). Hence, selecting growing animal models to study the interference of mistimed eating on intestinal microbiological diurnal rhythm is of great significance to fill knowledge gaps regarding the impact of irregular lifestyle on the health of children and growing animals.

Rabbits are widely used in clinical trials, and are also an important source of high-quality protein for humans (Bosze Zs and Houdebine, 2006; Cullere and Dalle Zotte, 2018). Similar to the irregular eating habits in humans, daytime eating in rabbits is contrary to their natural feeding habits and may disrupt the diurnal rhythm of the intestinal microbes, thereby affecting their accuracy in the study of intestinal diseases and productivity in rabbit breeding (Krohn et al., 1999; Gidenne et al., 2009; Guo et al., 2021). Previous studies have suggested that intestinal microbes play a critical role in maintaining the metabolic health, and that mistimed eating can cause elevated abundance of proinflammatory microbes and alterations in the composition of rhythmic microbes. This imbalance can disrupt the host metabolic homeostasis and activate inflammatory signaling pathways, ultimately promoting the occurrence of metabolic syndromes, chronic inflammation, and intestinal cancer (Asher and Sassone-Corsi, 2015; Deaver et al., 2018; Parkar et al., 2019). Recent studies revealed that occurrence of these diseases is associated with microbial metabolites-mediated signaling molecules that disrupt clock gene expression in peripheral tissues, such as the intestine and liver (Thaiss et al., 2016; Liang and FitzGerald, 2017). Currently, the microbial driving signal molecules, primarily including short-chain fatty acids (SCFAs), bile acids, hydrogen sulfide, vitamins, and serotonin, have been proven to regulate the expression of clock genes in peripheral tissues (Tahara et al., 2018; Frazier and Chang, 2020; Ku et al., 2020; Li et al., 2021). Among them, *Clostridium* species is one of the main microbial flora in the intestine that contributes for the synthesis of more than 95% of serotonin by intestinal chromaffin cells (Yano et al., 2015). Moreover, it was confirmed that additional serotonin *in vitro* could increase the diurnal expression of *PER1* and *BMAL1* in peripheral tissues and suprachiasmatic nucleus through G protein-coupled receptor signaling pathways, and also promote proliferation of intestinal epithelial cells (Paulus and Mintz, 2012; Aoki et al., 2014; Yano et al., 2015; Moon et al., 2020). Notably, intestinal epithelial cells are renewed every 4–5 days. When the diurnal rhythm of clock genes expression is disturbed, the proliferation of intestinal epithelial cells is blocked, resulting in the loss of intestinal barrier integrity (Stokes et al., 2017; Parasram and Karpowicz, 2019). In addition, tight junction genes that maintain intestinal barrier function are also regulated by *BMAL1* and *CLOCK*, exhibiting diurnal rhythms (Kyoko et al., 2014). Thus, unhealthy lifestyles, such as jet lag and a high-fat diet, can disrupt the diurnal rhythm of the intestinal barrier function, thereby increasing the susceptibility to intestinal diseases (Tuganbaev et al., 2020). However, further investigation is required to determine whether mistimed eating in growing rabbits disrupts the diurnal expression of the intestinal clock gene through microbial-driven serotonin signaling. Whether this leads to circadian disruption of the intestinal barrier function regulated by clock genes and decreased intestinal epithelial cell proliferation, ultimately causing an increased risk of intestinal diseases such as diarrhea, also requires investigation.

As a nonpharmacological intervention, time-restricted feeding to limit eating time and match the endogenous diurnal rhythm of the body is expected to correct diurnal rhythm disorders of peripheral tissue clock genes, and optimize intestinal flora composition and diurnal rhythm, thereby reducing the risk of inflammatory intestinal diseases (Hu et al., 2018; Ye et al., 2020). Current studies have shown that restricting the eating time window during the daytime activity phase of diurnal animals or nighttime activity period in nocturnal animals can promote metabolic health. Moreover, inappropriate time-restricted feeding regimes, such as alternate-day fasting, has no beneficial effect on regulating the metabolism or cardiovascular health, which may be related to the mismatch between the eating-fasting window and the endogenous diurnal rhythm (Hatori et al., 2012; de Cabo and Mattson, 2019; Waldman et al., 2020; Templeman et al., 2021). These results suggest that matching the window period of restricted eating occurring to the endogenous diurnal rhythm is key to determine the beneficial effects of time-restricted feeding. Furthermore, our previous research also determined that restricting the eating window in growing rabbits to occur at nighttime can match its rhythm of activity behavior, thereby enhancing the diurnal rhythm expression and amplitude of peripheral tissue clock genes (Guo et al., 2021). At the same time, restricted eating at night also strengthened the diurnal rhythm of body temperature by eating and activity behavior in growing rabbits. *In vitro* square wave temperature simulation experiments have confirmed that body temperature is an important zeitgeber, promoting cell proliferation and clock gene diurnal rhythm expression (Brown et al., 2002; Buhr et al., 2010; Guo et al., 2021). These results suggest that whether microbial-driven serotonin rhythm matches the eating activity-driven body temperature oscillations may be critical in influencing the intestinal health of growing rabbits. To confirm this hypothesis, in this study, growing rabbits were used as experimental model to explore whether night-restricted feeding can improve intestinal health through synchronization between microbial-driven serotonin rhythm and eating activity-driven body temperature oscillations through *in vitro* and *in vivo* experiments, thereby reducing the high risk of diarrhea in early developmental stages.

## Materials and Methods

### Ethics Statement

All animal management and experimental procedures were carried out in accordance with the Guidelines for Experimental Animals established by the Ministry of Science and Technology. All experimental protocols were approved by the Ethical Committee of China Agricultural University (AW31101202-1-2).

### Experimental Design and Sample Collection

**Figure 1:**
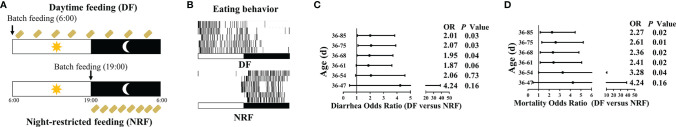
Experimental design and morbidity of growing rabbits (n = 216). **(A)** Design of animal feeding regimens. DF rabbits were allowed access to food throughout the day. NRF rabbits were allowed access to food from 19:00 PM to 6:00 AM. Yellow boxes indicate food availability. **(B)**The diurnal rhythm of eating behavior. **(C)** Odds ratio of diarrhea and **(D)** mortality risk in growing rabbits during the experiment. DF, daytime feeding; NRF, night-restricted feeding.

A total of 216 weaning female Ira rabbits (35 days of age, initial body weight 0.91 ± 0.10 kg) were raised in an open barn at the Qingdao Kangda Rabbit Development (Shandong, China) during the summer. According to our previous study (Guo et al., 2021), rabbits with similar body weight were randomly assigned to daytime feeding (DF, n = 108) and night-restricted feeding (NRF, n = 108) groups and these rabbits were housed and fed in individual cages (three rabbits per cage). The DF group could access food throughout the day (batch feeding at 6:00 AM; ZT: Zeitgeber time; ZT0 denotes sunrise at 6:00 AM). The NRF group could access food only at nighttime (from 19:00 PM to 6:00 AM; batch feeding at 19:00 PM, ZT13 denotes sunset at 19:00 PM) (**Figure 1A**). All rabbits were provided with water *ad libitum*. The same amount of food was provided to both groups; any surplus feed was cleared away and weighed daily, and there was no significant difference in food intake between the two groups throughout the experiment. The diet was antibiotic-free, and formulated to provide the predicted nutrient requirements of the growing rabbits according our previous study (Wang et al., 2019).

Three rabbits from each group were selected for observation eating behavior by software (The Observer XT, Wageningen, Netherlands). Six rabbits from each group were anesthetized and embedded thermometer in abdominal cavity (Star Oddi DST micro-T; MeterMall, OH, USA). In addition, mortality and diarrhea were recorded daily during the experiment. At 84 days of age, serum was collected from six healthy rabbits of each group and then immediately euthanized by cervical dislocation at 4-h intervals (daytime: 7:00, 11:00, and 15:00; nighttime: 19:00, 23:00, and 3:00). The intestinal content from the mid-cecum was collected and stored at −80°C for subsequent genomic DNA isolation and metabolites analysis. The middle segments of the jejunum and cecum were collected for the evaluation of gene expression, and the jejunum samples collected at 7:00 were used for examination of intestinal morphology.

### Microbial DNA Extraction and Sequencing

Genomic DNA was extracted from cecal contents using the Power Soil DNA Isolation Kit (MO BIO Laboratories, Carlsbad, CA, USA) following the manufacturer’s instructions. DNA was stored at −80°C until further processing. The V3−V4 region of the 16S rRNA gene with specific barcodes was amplified using the forward primer 5′-ACTCCTACGGGAGGCAGCA-3′ and the reverse primer 5′-GGACTACHVGGGTWTCTAAT-3′. After amplification, high-throughput sequencing was performed using the Illumina HiSeq 2500 PE250 platform (Illumina, San Diego, CA, USA). The raw sequencing data are available in Sequence Read Archive (SRA) with accession numbers (PRJNA632844).

### Bioinformatic Analysis

Raw reads were uploaded into Quantitative Insights into Microbial Ecology (QIIME2) software and the DADA2 software package wrapped in QIIME2 was used to quality filter, trim, denoise, and merge fastq files (Callahan et al., 2016; Bolyen et al., 2019). Clean reads were then conducted on feature classification to output amplicon sequence variants (ASVs) by DADA2, and the ASVs with relative abundance < 0.005% were filtered. Taxonomy annotation of the ASVs was performed based on the SILVA 132 database with naïve Bayes classifier. In addition, sequence data were rarefied to a depth of 29,675 sequences per sample for diversity calculations. For beta diversity analysis, nonmetric multidimensional scaling (NMDS) were performed according to unweighted UniFrac distances. Significant differences among groups were tested by analysis of similarity (ANOSIM). Furthermore, we employed Linear Discriminant Analysis (LDA) effect size to select biomarkers within different groups. SIMCA (version 13.0) was used to partial least-squares discriminant analysis (PLS-DA).

### SCFA and Metabolite Measurements

SCFAs were measured by gas chromatography according to a previously described method (Wang et al., 2019). Briefly, 1.5 g of thawed cecal content was resuspended in 1.5 mL of sterile distilled water, and the entire sample was centrifuged at 15,000 × g for 10 min at 4°C. The supernatant (1 mL) was collected, transferred to an ampule, and vortexed with 200 μL of metaphosphoric acid. The mixture was then incubated in an ice bath for 30 min. After centrifugation at 15,000 × g for 10 min, the sample was injected into a gas chromatograph (centrifugation at 15,000 × g) equipped with an HP 19091N-213 column (30.0 m × 0.32 mm; Agilent, Santa Clara, CA, USA). Injector and detector temperatures were 185°C and 210°C, respectively. In addition, non-starch polysaccharides in cecal content were quantified by liquid chromatography-tandem mass spectrometry (LC-MS/MS) using a Dionex Ultimate 3000 HPLC-system (AB Sciex, Darmstadt, Germany) connected to a tandem API 3200 Q Trap MS/MS device (AB Sciex) according to a previously described method (Evans et al., 2009).

### Intestinal Morphology

Jejunum specimens were immediately fixed in 4% (v/v) paraformaldehyde, and tissues were dehydrated and embedded according to standard procedures. Tissues embedded in paraffin blocks were sectioned (4 µm) and stained with hematoxylin and eosin (H&E). Based on the analysis of representative microscopic images, intestinal villus height and crypt depth were measured using NIS-Elements Basic Research software, version 2.20 (Nikon, Tokyo, Japan).

### Cell Culture and Assessment of Cell Proliferation

Rabbit intestinal epithelial cell (RIEC) was a kind gift from Prof. Dong-Sheng Che (Jilin Agricultural University). RIEC was cultured in DMEM/F12 (11330-032, Gibco, Carlsbad, CA, USA) medium with 5% serum and 1% penicillin/streptomycin. RIEC were incubated in 5% CO_2_ at 37°C. For serotonin (Ser) treatment, 10 μM serotonin (Sigma, St. Louis, MO, USA) was added to the medium at 37°C. Square-wave temperature (SWT) treatment according to our previous study (Guo et al., 2021), cells were cultured under a square-wave temperature rhythm (12 hours at 39°C, 12 hours at 35°C) for 4 days, the cells in control group were cultured at 37°C. For serotonin and body temperature matching treatment, cells were added with serotonin at 39°C (39°C + Ser) or 35°C (35°C + Ser) during square-wave temperature treatment. Cells were collected every 4 h for 24 h.

RIEC was seeded in 96-well plates at a density of 1 × 10^4^ cells/well. Cell viability of RIEC was measured every 12 h using the Cell Counting Kit-8 (CCK-8; Beyotime Institute of Biotechnology, Shanghai, China). After cell viability assay, the BeyoClick™ EdU cell proliferation kit (Beyotime, Shanghai, China) was used to detect RIEC proliferation following the manufacturer’s protocol. Briefly, 10 μM EdU was added to the wells for 2 h at 37°C. Then, cells were fixed and permeabilized with 4% para-formaldehyde and 0.3% Triton X-100 for 15 min at room temperature, respectively. After cells were washed with 3% BSA in PBS, cells were incubated with lick additive solution in dark for 30 min. RIEC nuclei were visualized with Hoechst 33342 (Olympus, Tokyo, Japan). At least five randomly separate fields from each sample image were used to quantification of RIEC proliferative rate.

### Real-Time Quantitative PCR Analysis (qPCR)

Total RNA was extracted from cecum, jejunum, and RIEC specimens using TRIzol reagent (Invitrogen, Carlsbad, CA, USA), and reverse transcription was performed according to the manufacturer’s protocols. Analysis of mRNA expression was performed using SsoFast EvaGreen Supermix in a CFX96 real-time PCR machine (Bio-Rad, Hercules, CA, USA). The relative mRNA expression of each group was normalized to glyceraldehyde-3-phosphate dehydrogenase (GAPDH) mRNA expression and data were analyzed using the 2^-ΔΔCT^ method. Primer pairs are listed in **Table S1**.

### Enzyme-Linked Immunosorbent Assay (ELISA)

Serum levels of interleukin (IL)-1β, IL-6, IL-10, IL-17, interferon (IFN)-γ, nuclear factor kappa β (NF-κβ), tumor necrosis factor (TNF)-α, lipopolysaccharide (LPS), and serotonin were determined using matched antibody pair enzyme-linked immunosorbent assay (ELISA) kits (R&D Inc., Beijing, China), according to the manufacturer’s instructions.

### Statistical Analysis

The non-parametric Jonckheere−Terpstra−Kendall Cycle (JTK_Cycle) was used to analyze the significance, amplitude, and phase of 24-h rhythms as previously described in R (Hughes et al., 2010). The odds ratio (OR) was used to evaluate the incidence of diarrhea and mortality, and the Chi-square test was calculated at a 95% confidence interval (95% CI). Differences in non-parametric data between the two groups were analyzed using the Wilcoxon rank-sum test. For all other data, Student’s *t*-test was used for comparing the differences between two groups. Statistical analyses were performed using SPSS 20.0 software (SPSS, Inc., Chicago, IL, USA). In addition, Spearman’s rho non- parametric correlations and *p*-values (false-discovery rate corrected *p* value) were calculated using the Psych packages (http://cran.r-project.org/web/packages/psych/). Figures were generated by Prism 7.0 software (GraphPad Software, Inc., La Jolla, CA, USA).

## Results

### NRF Enhances the Diurnal Oscillation of Body Temperature and Reduces the Risk of Diarrhea in Growing Rabbits

Analysis of the eating behavior of growing rabbits in the DF and NRF groups revealed that the DF group ate mainly during the daytime, whereas the eating behavior of the NRF group occurred only at nighttime (**Figure 1B**). Moreover, in agreement with our previous study (Guo et al., 2021), NRF was found to strengthen diurnal oscillations of body temperature by eating and activity behavior in growing rabbits and significantly increased body temperature during nighttime (**Supplementary Figure 1A**). In addition, the cumulative risk of diarrhea was also significantly lower in the NRF group after day 68 (**Figures 1C, D**; *p* < 0.05). These results indicate that NRF is consistent with nocturnal feeding habits in growing rabbits, which is more conducive to healthy breeding.

### NRF Significantly Alters the Structure and Composition of the Intestinal Microbiota in Growing Rabbits

**Figure 2:**
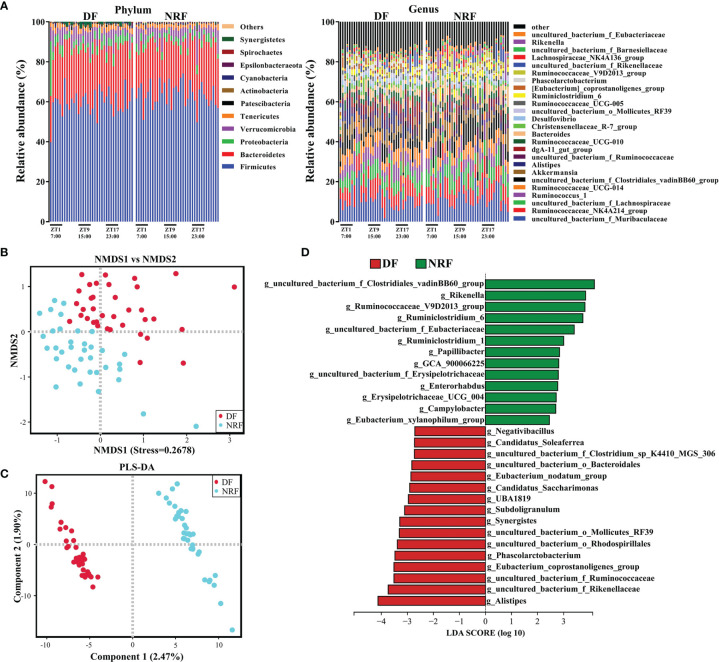
Feeding time affects the composition and microbial diversity of the gut microbiota in growing rabbits. **(A)** The taxonomic composition of cecum microorganisms at the phylum level and genus level (1%, according to relative abundance) under different feeding time regimens. **(B)** Nonmetric multidimensional scaling (NMDS) and **(C)** PLS-DA analysis of the cecal microbe. **(D)** LEfSe analysis at the genus level (LDA > 2). DF, daytime feeding; NRF, night-restricted feeding.

The dominant microbial phylum in growing rabbits was Firmicutes followed by Bacteroidetes, Verrucomicrobia, and Proteobacteria (**Figure 2A**). NMDS analyses (unweighted UniFrac) revealed that NRF and DF divided microorganisms into two distinct categories (**Figure 2B**). Further analysis by ANOSIM revealed that there was significant differences in microflora structure between the NRF and DF groups (**Supplementary Figure 1B**; R = 0.676, *P* = 0.002). Similar results were further confirmed by PLS-DA analysis (**Figure 2C**; R^2^ (Y) = 0.804, Q^2^ = 0.613), indicating a significant effect of the eating time on the gut microbiota structure.

To further explore the influence of NRF on the gut microbe composition of growing rabbits, linear discriminant analysis effect size analysis (LDA > 2) and Wilcoxon rank-sum test was performed on the gut microbiota of DF and NRF groups at different taxonomic levels. Firmicutes and Epsilonbacteraeota were enriched in the NRF group, whereas Proteobacteria, Synergistetes, and Patescibacteria were enriched in the DF group (**Supplementary Figure 1C**). At the genus level (**Figure 2D** and **Supplementary Table S2**), the NRF group was enriched with beneficial microorganisms, such as *Rikenella*, *Ruminococcaceae_V9D2013_group*, and *Ruminiclostridium_6*). In contrast, DF significantly enriched potentially conditioned pathogenic bacteria, such as *Synergistes*, *Desulfovibrio*, and *Alistipes* (Vartoukian et al., 2007; Rowan et al., 2010; Chen et al., 2017). These results indicate that NRF significantly changed the structure and optimized the composition of the intestinal microbiome in growing rabbits.

### NRF Optimizes the Rhythmic Microorganisms and Promotes the Production of Beneficial Metabolites in Growing Rabbit

**Figure 3:**
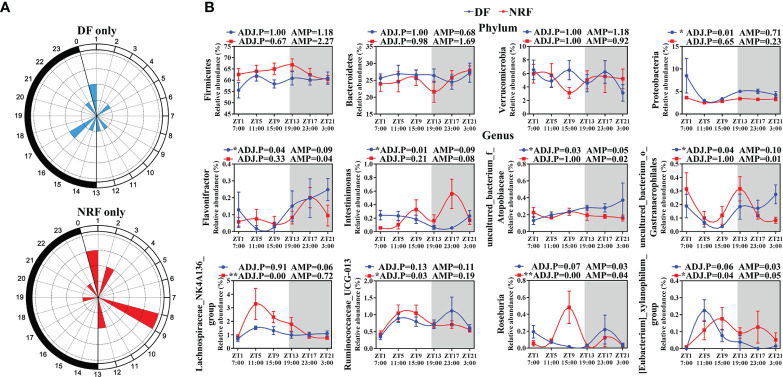
The diurnal rhythm of gut microbiome phyla and genus under different feeding time regimens. **(A)** Polar plot represents the number of rhythmic ASVs with an estimated peak value for each time as determined by JTK analysis. The radius of black concentric circles indicates the number of rhythmic ASVs, and the minimum radius of the black concentric circle represents one ASV. The black arc on the left side of the polar plot indicates the day/night cycle. **(B)** The diurnal rhythm of gut microbiome at phylum and genus level (n = 6 per time point), data are shown as the mean ± SEM. White boxes indicate daytime and gray boxes indicate nighttime. ADJ.P for adjusted minimal *p*-values, ADJ.P < 0.05 indicates significant diurnal rhythm, AMP represents amplitude. DF, daytime feeding; NRF, night-restricted feeding. *ADJ.P < 0.05; **ADJ.P < 0.01.

To assess the 24-h diurnal rhythm variation of gut microorganisms in growing rabbits, the ASVs in the DF and NRF groups were evaluated by JTK analysis. Approximately 3% and 4% of ASVs in DF and NRF groups were found to have a diurnal rhythm (**Supplementary Figure 2A**; ADJ.P < 0.05), respectively. Venn diagram analyses further demonstrated that the rhythmic ASVs were specific for the DF and NRF groups (**Supplementary Figure 2B**). Radar plot also showed that the ASVs rhythmic peaks in the DF group were distributed across all time points, whereas in the NRF group occurred primarily during the daytime fasting periods (**Figure 3A**). Subsequently, JTK analysis of the microorganisms at the phylum level determined that DF caused a diurnal rhythm in Proteobacteria as compared with the NRF group (**Figure 3B**; ADJ.P < 0.05). At the genus level, *Intestinimonas* and *Flavonifractor* showed diurnal rhythm in the DF group (**Figure 3B** and **Supplementary Table S3**). In contrast, NRF promoted diurnal rhythm of *Clostridium* (*Lachnospiraceae_NK4A136_group*, *Ruminococcaceae_UCG-013*, and *Roseburia*), which are related to promoting the synthesis of serotonin by intestinal chromaffin cells (Yano et al., 2015; Mandic et al., 2019). These results indicated that NRF reprogrammed the diurnal rhythm of gut microbiota.

**Figure 4:**
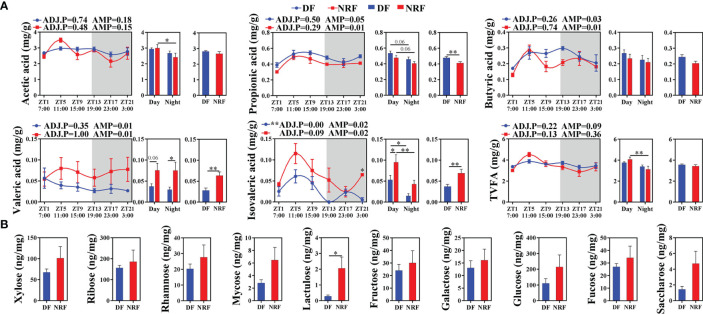
Effects of feeding time on the metabolites of cecal microbiome in growing rabbits. **(A)** Effects of feeding time on the concentration of short-chain fatty acids in cecum (n = 6 per time point). White boxes indicate daytime and gray boxes indicate nighttime. ADJ.P for adjusted minimal *p*-values, ADJ.P < 0.05 indicates significant diurnal rhythm, AMP represents amplitude, *ADJ.P < 0.05; **ADJ.P < 0.01. **(B)** Effects of feeding time on sugar metabolites in cecum (n = 6). Differences between the DF and NRF groups were determined with a t-test, *p < 0.05. Data are shown as the mean ± SEM. DF, daytime feeding; NRF, night-restricted feeding.

Moreover, NRF-induced gut microbiota structure and composition changes in growing rabbits were found to be accompanied by changes in microbial metabolites. JTK analysis of SCFA concentrations in cecal contents revealed that only isovaleric acid followed a diurnal rhythm in the DF group (**Figure 4A**; ADJ.P < 0.05). The mean propionic acid concentration in the DF group was significantly higher than in the NRF group across all times points, whereas valeric acid and isovaleric acid concentrations in the NRF group were significantly higher than those in the DF group (*p* < 0.01). In addition, analysis of non-starch polysaccharides in cecal contents revealed that NRF resulted in significantly higher concentrations of lactulose (**Figure 4B**). Taken together, NRF promoted diurnal rhythm changes in beneficial intestinal microorganisms and the production of beneficial metabolites.

### NRF Significantly Enhances the Rhythmic Expression of Intestinal Clock Genes and Clock-Controlled Genes in Growing Rabbits

**Figure 5:**
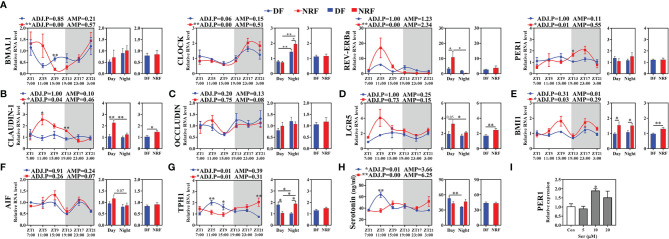
Effects of feeding time on the expression of clock and clock-controlled genes in cecum of growing rabbits (n = 6 per time point). **(A)** The expression of clock genes. **(B, C)** Tight junction genes. **(D–F)** Intestinal epithelial cells regeneration and apoptosis genes. **(G, H)** Serotonin synthesis rate-limiting enzyme *TPH1* genes expression and the diurnal rhythm of serotonin in serum. White boxes indicate daytime and gray boxes indicate nighttime. **(I)** Expression of *PER1* gene in rabbit intestinal epithelial cells induced by serotonin. ADJ.P for adjusted minimal *p*-values, ADJ.P < 0.05 indicates significant diurnal rhythm, AMP represents amplitude, *ADJ.P < 0.05; **ADJ.P < 0.01. Differences between the DF and NRF groups were determined with a t-test, *p < 0.05; **p < 0.01. DF, daytime feeding; NRF, night-restricted feeding. Data are shown as the mean ± SEM.

Microbial-driven metabolite signals regulate the expression of host peripheral tissue clock genes. Therefore, the effects of NRF on the intestinal clock gene expression in growing rabbits was explored. JTK analysis of the intestinal clock gene expression revealed that NRF significantly promoted the diurnal rhythm expression and amplitude of *BMAL1*, *CLOCK*, *REV-ERBα*, and *PER1* in the cecum compared with the expression patterns observed in the DF group (**Figure 5A**; ADJ.P < 0.05). Subsequent analysis of the expression of *CLAUDIN-1*, a key regulator of the intestinal barrier function, revealed that NRF promoted the high-in-daytime and low-at-night rhythm (ADJ.P < 0.05). Moreover, NRF increased the average expression of *CLAUDIN-1* (**Figure 5B**; *p* < 0.05), but the level of *OCCLUDIN* was no significant difference between the two groups (**Figure 5C**). The proliferation and renewal of intestinal epithelial cells is essential to maintain the integrity of the intestinal mucosa, a process that is regulated by the proliferation and differentiation of crypt stem cells mediated by *BMI1* and *LGR5*. Herein, the expression of *BMI1* was found to have a diurnal rhythm in the NRF group (ADJ.P < 0.05), and the expression of both *BMI1* and *LGR5* was significantly higher than that in the DF group throughout the day (*p* < 0.01), whereas no significant difference was identified between the two groups for apoptosis inducing factors (**Figures 5D–F**). Similar to cecum results, NRF also promoted the diurnal rhythm expression of *BMI1* and *CLAUDIN-1* in the jejunum (**Supplementary Figures 3A–E**). Therefore, whether NRF affected the intestinal morphology in growing rabbits was evaluated next. Unlike the cecum, the mucosa of the jejunum is covered with villi, which makes it relatively easy to detect changes in intestinal morphology. Overall, NRF was found to significantly increase the height of jejunal villi (*p* < 0.05), but had no significant effect on the depth of the intestinal crypts and mucosal thickness (**Supplementary Figure 3F**). Therefore, these results suggest that NRF strengthens the diurnal rhythm of intestinal clock genes and clock-control genes, which is partly beneficial for promoting intestinal barrier function and integrity in growing rabbits.

The immune function of the body is regulated by clock genes and also changes in diurnal rhythm (Murakami and Tognini, 2019). To investigate the effect of NRF on the immune function in growing rabbits, immune-related indicators in the serum of growing rabbits were analyzed. NRF was found to significantly increase the diurnal amplitude of IL-10 and decreased the diurnal amplitude of NF-κβ, IL-1β, IL-6, TNFα, and IFN-γ in the serum (**Supplementary Figure 4A**). Moreover, it significantly reduced IL-6 concentrations during the nighttime period (*p* < 0.05). Next, analyses at the gene level also showed that NRF reduced the diurnal amplitude of *NF-κβ* and increased the diurnal amplitude of *IL-10* (**Supplementary Figure 4B**). Therefore, these results suggest that NRF reduced the diurnal amplitude and concentration of proinflammatory factors that may be related to the reduction of host susceptibility to disease (Hilker and Schmitz, 2008).

### Diurnal Oscillations of Serotonin and Body Temperature Induced by NRF Can Regulate Diurnal Rhythmic Expression of Intestinal Clock Genes

Microbial-driven metabolite signals and body temperature diurnal oscillations can both regulate the diurnal rhythm expression of clock genes in peripheral tissues (Buhr et al., 2010; Leone et al., 2015; Luzader et al., 2018). To further explore the regulatory relationship between host intestinal clock genes, gut microbiota, and body temperature oscillations, correlations between ASVs, clock genes, clock controlled genes, microbial metabolites, and body temperature diurnal oscillation were evaluated in the NRF or DF group. *Ruminococcaceae* spp. belonging to the *Clostridium* family were found to regulate the synthesis of serotonin in intestinal chromaffin cells. In the NRF group, *Ruminococcaceae_NK4A214_group* was found to be positively correlated with serotonin synthesis rate limiting enzymes tryptophan hydroxylase isoform 1 (*TPH1*) and *CLOCK* (**Supplementary Figure 5A**). Moreover, NRF showed to promote the rhythmic expression of *TPH1*, and the serum serotonin concentration also showed the low-in-daytime and high-at-night rhythm (**Figures 5G, H** **)**, which was consistent with the diurnal rhythm of body temperature in growing rabbits. However, *TPH1* expression and peak of serotonin concentration in the DF group were detected during daytime, which was contrary to the peak of body temperature in growing rabbits at nighttime. Furthermore, a negative correlation between body temperature and serotonin in the DF group was observed (**Supplementary Figure 5D**), whereas a positive correlation was identified between body temperature and *TPH1* and *CLOCK* in the NRF group (**Supplementary Figure 5B**). These results imply that the synchronization of microbial-driven serotonin rhythm and eating activity-driven body temperature oscillations may be critical for NRF to alter the rhythmic expression of intestinal clock and tight junction genes.

**Figure 6:**
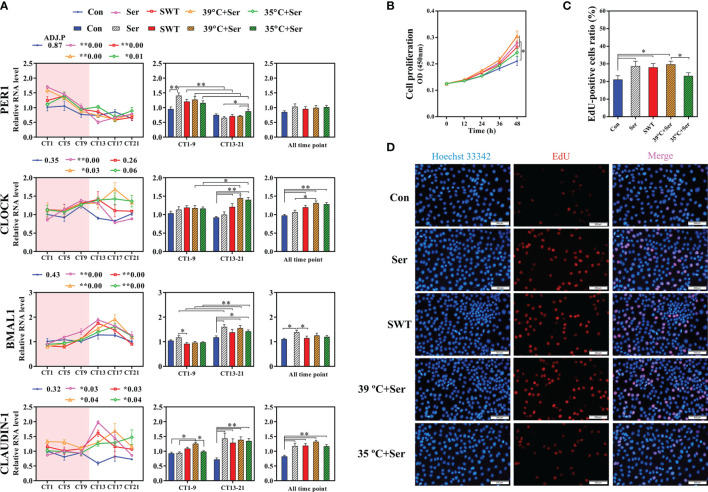
Effects of serotonin and temperature diurnal oscillation on expression of clock and tight junction gene in rabbit intestinal epithelial cells (RIEC). **(A)** Effects of rhythmic addition of serotonin and square wave temperature on the expression of clock and Claudin-1 in RIEC. The control group (Control, Con); the 12 h rhythmically added serotonin group (Serotonin, Ser); the square wave temperature group (SWT); addition of serotonin at 39°C (39°C + Ser); addition of serotonin at 35°C (35°C + Ser). **(B)** Cell viability of RIEC. **(C)** The percentage EdU-positive RIECs and **(D)** RIEC proliferation using EdU staining. ADJ.P for adjusted minimal *p*-values, ADJ.P < 0.05 indicates significant diurnal rhythm, AMP represents amplitude, *ADJ.P < 0.05; **ADJ.P < 0.01. Differences between the groups were determined with a t-test. Data are shown as the mean ± SEM. *p < 0.05; **p < 0.01.

To further verify this hypothesis *in vitro*, simulation of the rhythmic synchronization of serotonin and square wave temperature were respectively used to detect the expression of clock and cell proliferation in RIEC. The dose addition of serotonin concentration to RIEC was tested, revealing that 10 μM serotonin significantly promoted *PER1* expression (**Figure 5I**). After 12-h rhythmic addition of 10 μM serotonin or 39–35°C square wave temperature treatment (SWT), the diurnal rhythm expression of *PER1*, *BMAL1*, *CLOCK*, and *CLAUDIN-1* were significantly increased (**Figures 6A**). Next, the rhythmic addition time of serotonin was performed during the high temperature period (39°C + Ser) to simulate the synchronization of serotonin rhythm and eating activity-driven body temperature oscillations in growing rabbits. *BMAL1*, *CLOCK*, and *CLAUDIN-1* showed significant rhythmic expression (**Figures 6A**), resulting in significantly higher expression of *CLAUDIN-1* than in the other groups in the CT1–CT9 time period (**Figure 6A**; *p* < 0.05), and also delayed the phases of *CLOCK* (**Supplementary Figure 6**). Afterwards, the rhythmic addition of serotonin in the low temperature period (35°C + Ser) was used to simulate the desynchronization of serotonin rhythm and eating activity-driven body temperature oscillations in growing rabbits. The diurnal amplitude of *PER1*, *BMAL1*, and *CLAUDIN-1* were significantly decreased (**Supplementary Figure 6**; *p* < 0.05). In addition, the proliferation of RIEC in the 35°C + Ser treatment group was significantly lower than that in the other three treatment groups (**Figures 6B–D**; *p* < 0.05). Therefore, these results suggest that NRF promotes the synchronization of serotonin rhythm and body temperature oscillations in growing rabbits, which is beneficial to strengthen the diurnal expression of intestinal clock gene and also promote proliferation of intestinal epithelial cells.

## Discussion

The endogenous diurnal rhythm of the body is regulated by multiple environmental factors (Zhang et al., 2020). Numerous studies have demonstrated that microbial-driven serotonin and body temperature oscillation, as important zeitgebers, participate in the regulation of the clock genes expression in peripheral tissues (Buhr et al., 2010; Leone et al., 2015; Luzader et al., 2018). However, most of the current studies have focused on the effects of single factors on clock gene expression of peripheral tissues or cells. Thus, it remains unclear whether the synchronization of microbe-driven serotonin rhythm and eating activity-driven body temperature oscillations improve gut clock gene expression. The present study reveals for the first time that NRF promotes diurnal rhythmic expression of intestinal clock and tight junction genes in growing rabbits *via* the synchronization of microbe-driven serotonin rhythm and eating activity-driven body temperature oscillations. In addition, NRF was shown to increase the abundance and diurnal rhythm of beneficial microorganisms, and enhance the production of beneficial metabolites. Moreover, NRF can also reduce the abundance of conditional pathogens and the diurnal amplitude of proinflammatory factors, thereby reducing the risk of diarrhea in growing rabbits.

Feeding and fasting cycles can cause diurnal fluctuations of indigenous spore-forming microbes (mainly *Clostridium*) (Thaiss et al., 2016; Liang and FitzGerald, 2017), thereby intervening in the process of serotonin synthesis by *Clostridium*-mediated intestinal chromaffin cells (Paulus and Mintz, 2012; Yano et al., 2015; Mandic et al., 2019). NRF was found to promote the diurnal rhythm of the gut microorganisms (such as *Lachnospiraceae_NK4A136_group* and *Ruminococcaceae_UCG-013*) belonging to *Clostridium*, and also promote the low-in-daytime and high-at-night rhythm of *TPHI*, which is a rate-limiting step in the biosynthesis of serotonin. In addition, the gut can synthesize more than 90% of the serotonin of the body, which is consistent with the diurnal rhythm of serotonin in the blood (Ebert-Zavos et al., 2013). For this reason, the concentration of serotonin in the serum was also herein evaluated. Overall, serotonin concentrations in the NRF group were found to exhibit low-in-daytime and high-at-night rhythm, whereas DF was associated with high-in-daytime and low-at-night rhythm, which may be related to serotonin being a derivative of tryptophan, an essential amino acid primarily derived from food (Pontes et al., 2010). These results suggest that microbial-driven serotonin synthesis in intestinal chromaffin cells are influenced by eating rhythms. Therefore, the rhythm of eating behavior in growing rabbits was analyzed, revealing that the eating time in the NRF and DF groups occurred at nighttime and daytime, respectively, which was consistent with the serotonin synthesis rhythm.

Serotonin, as an important zeitgeber, can regulate the expression of clock genes in peripheral tissues. Studies have reported that adding supernatant containing *Clostridium* metabolites to small intestinal organoids can induce changes in the diurnal rhythm and phase of *PER1* and *BMAL1* (Luzader et al., 2018; Ku et al., 2020). Herein, consistent with previous studies, NRF promoted serotonin diurnal rhythm, and strengthened the diurnal rhythm expression and amplitude of intestinal *BMAL1*, *CLOCK*, and *REV-ERBα*. Moreover, previous study confirmed that the CLAUDIN-1 regulated the intestinal barrier function through the interaction of the BMAL1–CLOCK heterodimer with its E-box promoter sequence (Kyoko et al., 2014). In addition, *LGR5* and *BMI1*, which control the proliferation and renewal of intestinal epithelial cells, were also regulated by BMAL1. However, mistimed eating times can lead to disordered diurnal expression of these genes, thereby increasing host intestinal permeability, and the risk of inflammatory bowel disease and metabolic disease (Stokes et al., 2017). Interestingly, NRF was found to enhance the expression of intestinal clock genes and promote the diurnal rhythm expression of *CLAUDIN-1* and *BMI1*, also increasing the expression of *CLAUDIN-1* and *BMI1* at the overall level throughout the day. These results suggest that NRF may promote diurnal expression of intestinal clock genes and downstream clock-controlled genes through microbial-driven serotonin in growing rabbits. To further verify this hypothesis, a rhythmic addition of serotonin *in vitro* experiment was performed to reveal that serotonin can induce the diurnal expression of clock and tight junction genes in RIEC, and promote cell proliferation, which was consistent with previous findings (Aoki et al., 2014; Spohn et al., 2016). Thus, these results partially confirm that microbial-driven serotonin signals, as one of the zeitgebers, regulate clock gene expression in growing rabbit intestinal epithelial cells.

The endogenous diurnal rhythms are regulated by multiple environmental factors to maintain the stability of the biological clock of the body (Zhang et al., 2020). Our previous research showed that NRF promotes the rhythms of eating and activity behavior to match the endogenous biological rhythms in growing rabbits, thereby increasing the diurnal oscillations of their body temperature (Guo et al., 2021). Body temperature, as an important zeitgeber, can activate cAMP-response element binding protein (CREB) through the Ca^2+^-CaM signaling pathway mediated by the temperature sensor TRPV4, thereby binding to the cAMP response element (CRE) located at the *PER1/2* promoter and initiates gene expression (Buhr et al., 2010; Shibasaki et al., 2015). Similarly, serotonin activates CREB through G protein-coupled receptor-mediated cAMP-PKA signaling pathway, thereby initiating *PER1/2* expression (Noda et al., 2004; Lee et al., 2010; Aoki et al., 2014). These results suggest that whether the microbial-driven serotonin is synchronized with the diurnal oscillations of body temperature may influence diurnal rhythmic expression of intestinal clock genes. The present study showed that the diurnal rhythm of serotonin in the NRF group was synchronized with body temperature oscillations. Serotonin peaks appeared in the eating and activity time of growing rabbits at nighttime, whereas those in the DF group appeared during the daytime, which may disrupt the expression of the clock gene as it is not synchronized with the diurnal oscillations of body temperature. Moreover, DF could lead to the loss of diurnal rhythm of cecal clock gene and *CLAUDIN-1*, and decrease the amplitude of these genes. Indeed, *in vitro* simulations of serotonin and body temperature asynchrony were found to decrease the diurnal amplitude of the RIEC clock gene and *CLAUDIN-1*, whereas simulations of serotonin rhythm synchronization with body temperature oscillations were found to enhance the diurnal amplitude of these genes. Therefore, these results partially confirm that microbial-driven serotonin rhythm and eating activity-driven body temperature oscillations synergistically promote the diurnal rhythm expression of gut clock gene and *CLAUDIN-1*. However, these findings raise new scientific questions, such as what type of relationship exists between microorganisms and body temperature oscillations? It has been shown that diurnal oscillations in body temperature cause changes in the diurnal rhythms of gut microbiota, and that gut microbiota can participate in host thermoregulation through the metabolites, such as SCFAs (Bo et al., 2019; Paulose et al., 2019). These results imply that there is an interaction between gut microbes and body temperature, which needs to be experimentally investigated using sterile animals, fecal bacteria transplantations, and TRPV-family members knockout models.

The intestinal epithelial barrier plays an important role in inhibiting bacterial translocation. When the intestinal barrier is impaired, it causes microbial translocation and subsequent activation of the innate immune system, thereby resulting in intestinal inflammatory diseases (Andersen et al., 2017; Yang et al., 2019). However, most of the current studies on intestinal barrier function have focused on the comparison of a single time point. Recent studies have found that tight junction genes, which play a critical role in the intestinal barrier, show diurnal rhythm, while simulating human shifts, jet lag, and high-fat diets were found to cause loss of the diurnal rhythms of intestinal tight junction genes in mice, leading to an increased risk of infestation by intestinal conditional pathogens (Kyoko et al., 2014; Tuganbaev et al., 2020). In the present study, both *in vivo* and *in vitro* experiments revealed that NRF synchronizes the microbial-driven serotonin rhythm and eating activity-driven body temperature oscillations, which promoted the diurnal rhythm expression and amplitude of tight junction genes, and also promoted the proliferation of RIEC. These results imply that NRF strengthens the intestinal barrier function in growing rabbits and helps prevent the invasion of pathogenic microorganisms. Subsequent analysis of microbiome data further revealed that DF significantly increases the relative abundance of *Synergistes* associated with an intestinal micro-ecological imbalance (Vartoukian et al., 2007; Bjorkhaug et al., 2019). In addition, *Desulfovibrio*, *Synergistes*, and *Alistipes* are potential pathogens, and their abundance significantly increased in the DF group. It is worth noting that an increase in *Desulfovibrio* abundance is a precursor to intestinal micro-ecological imbalances caused by the cytotoxic sulfide produced by this microorganism. Furthermore, cytotoxic sulfide can inhibit phagocytosis and bactericidal effects, as well as mitochondrial oxidative phosphorylation component cytochrome c oxidase, thereby causing ulcerative colitis and inflammatory bowel disease (Fite et al., 2004; Rowan et al., 2010; Nicholls et al., 2013). Thus, these results suggest that DF significantly increases the relative abundance of conditioned pathogenic bacteria in growing rabbits and increases the risk of intestinal inflammation, which is consistent with the findings in the current study where DF increased the rhythmic amplitude and concentration of proinflammatory factors in the intestine and serum. In contrast, NRF was able to promote the diurnal rhythm and relative abundance of microorganisms (such as *Ruminiclostridium* spp. and *Lachnospiraceae_NK4A136_group*) associated with the production of SCFAs, which could in turn inhibit histone deacetylases of macrophage and dendritic cells to reduce the expression of proinflammatory factors (Glauben et al., 2006; Fellows et al., 2018; Yuille et al., 2018). Herein, NRF also significantly increased the concentrations of valeric acid and isovaleric acids in the cecum, significantly decreased the concentrations of serum proinflammatory factors at some time points, and also decreased the rhythmic amplitude of proinflammatory factors, events that may be related to reduced host susceptibility to disease (Hilker and Schmitz, 2008). In addition, NRF increased the lactulose content in the cecum, which is a nondigestible prebiotic in the intestine that can promote intestinal health by regulating the growth of endophytic bacteria (Masanetz et al., 2011). Therefore, these results suggest that NRF matching with endogenous diurnal rhythms in growing rabbits strengthens the diurnal rhythm of intestinal clock genes and barrier function, optimizes the structure and composition of gut microbiota, and produces more beneficial metabolites to reduce concentration and diurnal amplitude of inflammatory cytokines, thereby reducing susceptibility to disease. Overall, these effects are consistent with NRF potential for reducing the risk of diarrhea in growing rabbits. However, the present study also has limitations. More detailed clock knockout and sterile animal experimental investigations are needed to determine the specific underlying molecular mechanisms of NRF for promoting intestinal health in growing rabbits.

In conclusion, showed for the first time that NRF reprograms the diurnal rhythm of the gut microbiome and promotes the synchronization of microbe-driven serotonin rhythm and eating activity-driven body temperature oscillations in growing rabbits. These events result in improved diurnal expression of intestinal clock genes and genes related to maintenance of intestinal barrier function, as well as in enhanced cell proliferation and renewal. Furthermore, through *in vitro* simulation experiments, serotonin rhythm was shown to synchronize with diurnal oscillations of body temperature, thereby promoting rhythmic expression of clock and tight junction genes in RIEC, and improving cell proliferation. Taken together, these results provide new perspectives to guide precision breeding of young animals and healthy eating habits in children.

## Data Availability Statement

The datasets presented in this study can be found in online repositories. The names of the repository/repositories and accession number(s) can be found in the article/**Supplementary Material**.

## Ethics Statement

All animal management and experimental procedures were carried out in accordance with the Guidelines for Experimental Animals established by the Ministry of Science and Technology. All experimental protocols were approved by the Ethical Committee of China Agricultural University (AW31101202-1-2).

## Author Contributions

Q-JW, YG, K-HZ, LZ, and S-XG performed experiments for the paper. Q-JW, C-HS, PL, M-QZ, Q-YJ, and Z-YL assisted in the analysis and data collection. M-YL, ML, and M-ZW interpreted the data. LA, J-HT, and Z-HW provided a critical revision of the manuscript. Z-HW conceived the original idea and the funding project. All authors contributed to the article and approved the submitted version.

## Funding

This research was supported by China Agriculture Research System of MOF and MARA (CARS-43-D-1) and Development Program of China (2016YFD0500506).

## Conflict of Interest

The authors declare that the research was conducted in the absence of any commercial or financial relationships that could be construed as a potential conflict of interest.

## Publisher’s Note

All claims expressed in this article are solely those of the authors and do not necessarily represent those of their affiliated organizations, or those of the publisher, the editors and the reviewers. Any product that may be evaluated in this article, or claim that may be made by its manufacturer, is not guaranteed or endorsed by the publisher.
