# Supplementary material for: Night-Restricted Feeding Improves Gut Health by Synchronizing Microbe-Driven Serotonin Rhythm and Eating Activity-Driven Body Temperature Oscillations in Growing Rabbits
Source: Front Cell Infect Microbiol. 2021 Dec 17;11:771088. doi: 10.3389/fcimb.2021.771088 (PMC8718905; doi:10.3389/fcimb.2021.771088)
Supplement: Supplementary file 1 [file Table_1.docx]

Supplementary Material

**Supplementary Figures and Tables**

**Supplementary** **Figures**


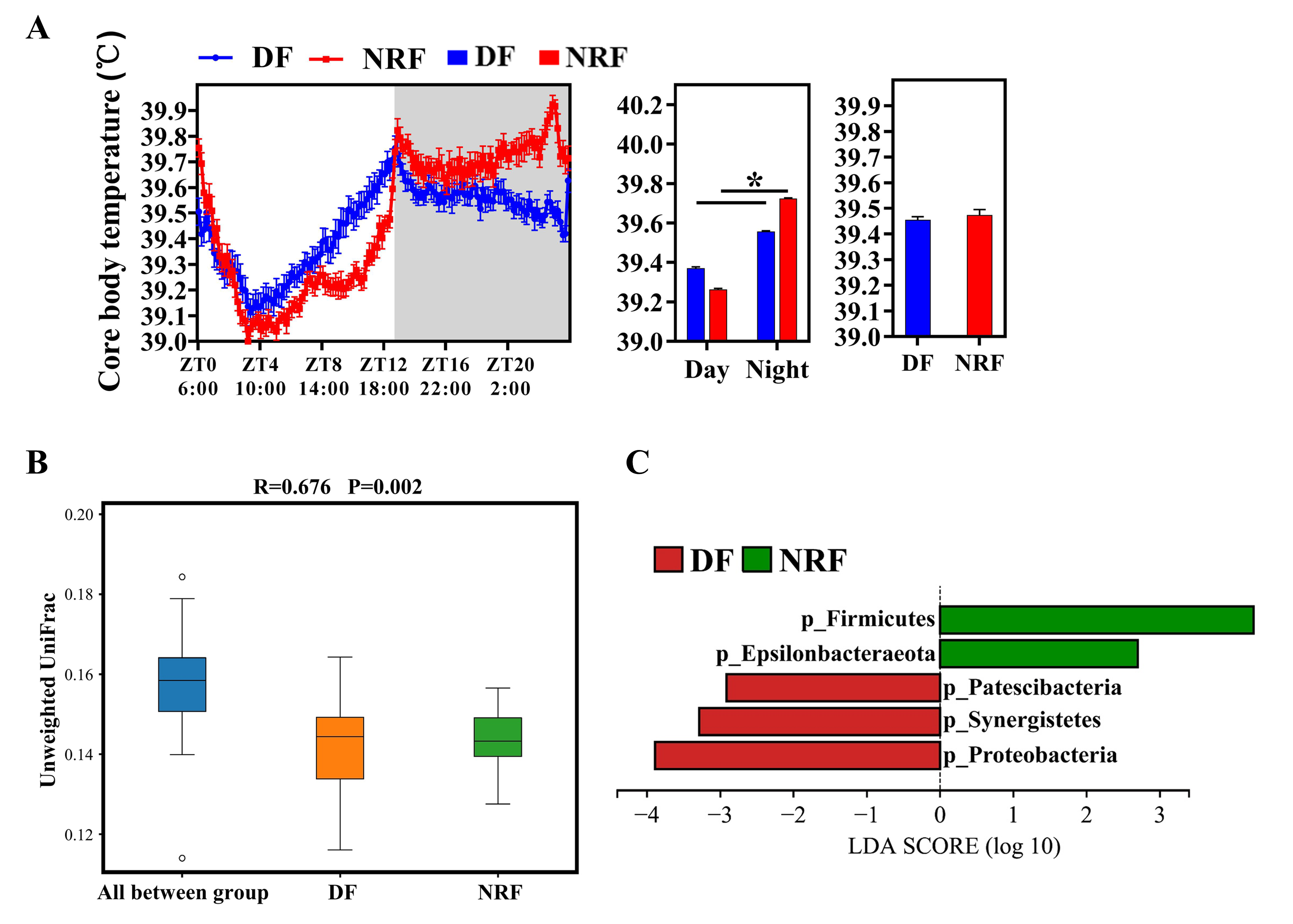


**Figure S1** Effects of feeding time on body temperature oscillation and the composition and diversity of the gut microbiota in growing rabbits. (A) Feeding time changes body temperature oscillation under DF and NRF feeding conditions. Results from our previous study were replicated (Guo et al., 2020). (B) ANOSIM analysis of cecum microbe. (C) LEfSe analysis at the phylum level (LDA > 2). DF, daytime feeding; NRF, night-restricted feeding.


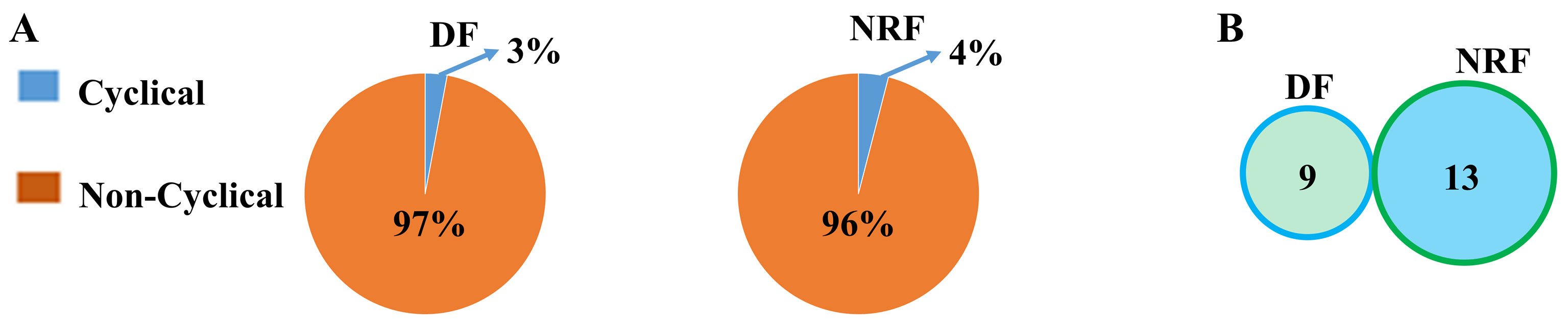


**Figure S2** Feeding time changes the number of rhythmic ASVs in growing rabbits. (A) Pie chart showing the percentage of rhythmic and non-rhythmic amplicon sequence variants (ASVs) in growing rabbits (n = 36). (B)The Venn diagram showing the number of ASVs followed a diurnal rhythm. DF, daytime feeding; NRF, night-restricted feeding.

**
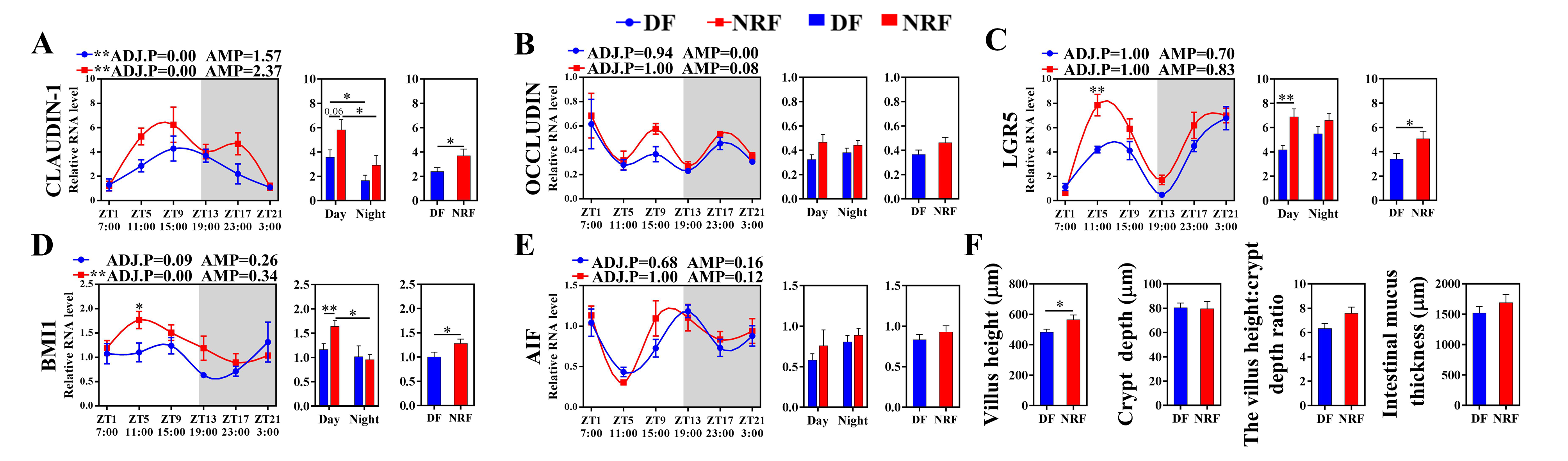
**

**Figure S3** Effects of feeding time on the expression of clock-controlled genes in jejunum of growing rabbits (n = 6 per time point). (A–B) Tight junction genes. (C–E) Intestinal epithelial cells regeneration and apoptosis genes. (F) The villus height, crypt depth, and intestinal mucus thickness in jejunum (n = 4). ADJ.P for adjusted minimal *p*-values, ADJ.P < 0.05 indicates significant circadian rhythm, AMP represents amplitude. Differences between the DF and NRF groups were determined with a t-test. DF, daytime feeding; NRF, night-restricted feeding. Data are shown as the mean ± SEM. * *p* < 0.05; ** *p* < 0.01.


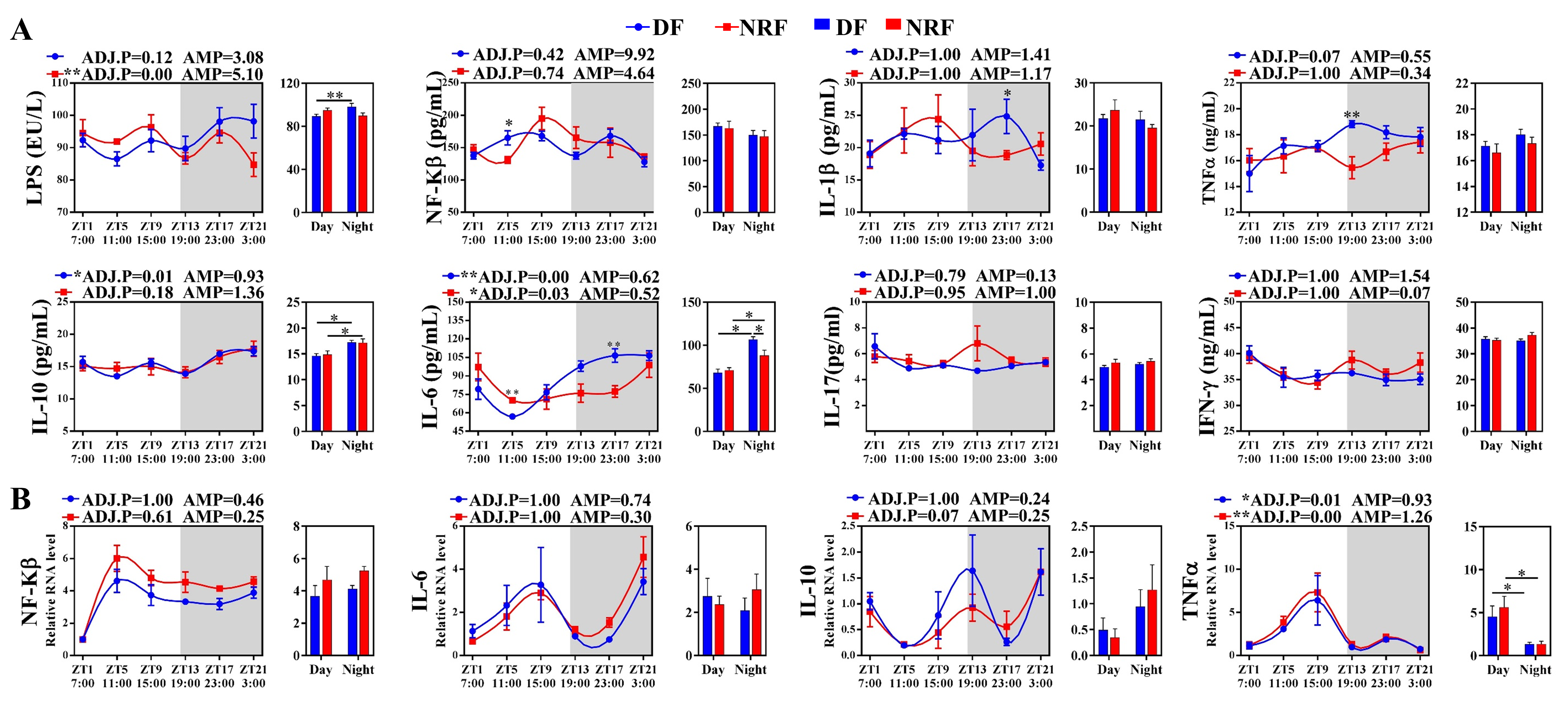


**Figure S4** Effects of feeding methods on cytokines in growing rabbits. (A) Effects of feeding methods on cytokines and LPS in serum of growing rabbits. (B) Effects of feeding time on the gene expression of cytokines in cecum of growing rabbits. n = 6 per time point. ADJ.P for adjusted minimal *p*-values, ADJ.P < 0.05 indicates significant circadian rhythm, AMP represents amplitude. Differences between the DF and NRF groups were determined with a t-test. DF, daytime feeding; NRF, night- restricted feeding. Data are shown as the mean ± SEM. * *p* < 0.05; ** *p* < 0.01.


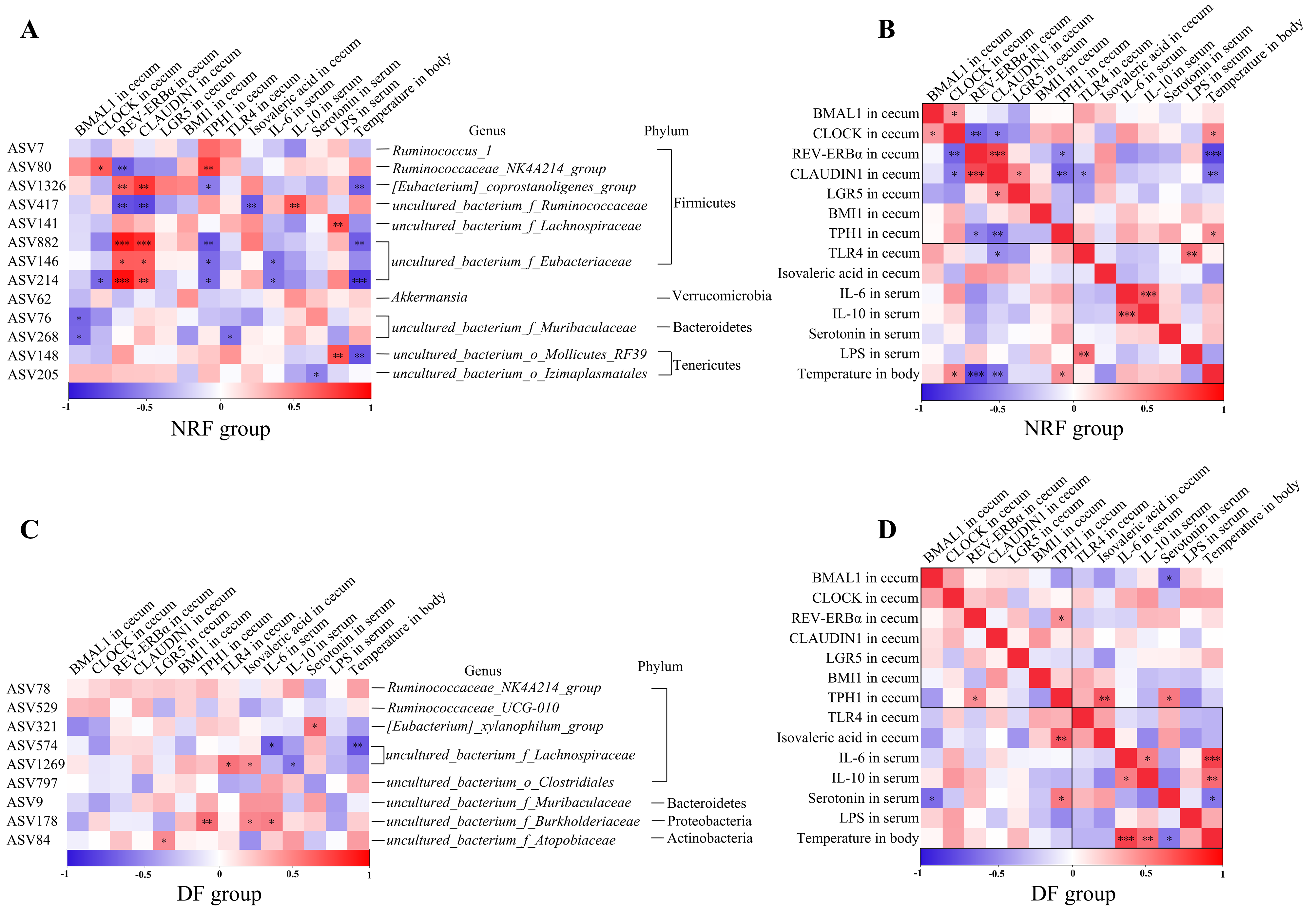


**Figure S5** Correlations between amplicon sequence variants (ASVs), serum immune factors, and cecum-related indices. (A) Correlation between rhythmic ASVs and serum immune factors or cecum-related indices in the NRF group. (B) Correlation between serum immune factors and cecum-related indices in the NRF group. (C) Correlation between rhythmic ASVs and serum immune factors or cecum-related indices in the DF group. (D) Correlation between serum immune factors and cecum-related indices in the DF group. Colors red and blue denote positive and negative correlation, respectively. Spearman’s rho non-parametric correlation was applied. DF, daytime feeding; NRF, night-restricted feeding. * *p* < 0.05; ** *p* < 0.01; *** *p* < 0.001.

**
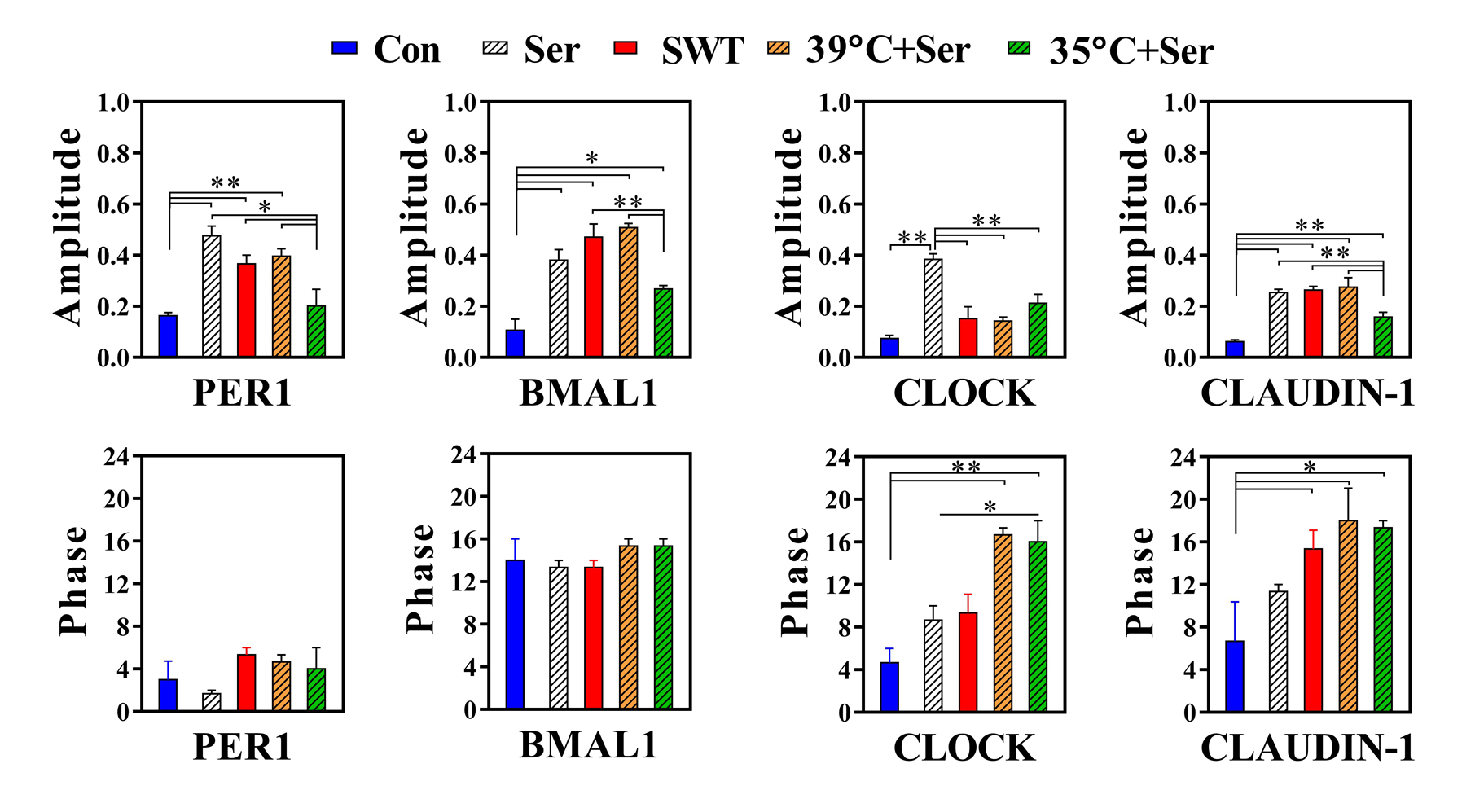
**

**Figure S6** JTK analysis the amplitude and phase of genes expression in rabbit intestinal epithelial cells. Data are shown as the mean ± SEM. * *p* < 0.05; ** *p* < 0.01.

**Supplementary Tables**

**Table S1** Primers used for qPCR

| Gene | Forward primer(5' to 3') | Reverse primer(5' to 3') |
| --- | --- | --- |
| *BMAL1* | GGGCTGGATGAAGACAACGA | CTAGGAGTTCCTGCGGCAAA |
| *CLOCK* | CACAGCGCAGCACTTGATAC | AAGCGAGGTTTGCTGACTGT |
| *PER1* | AGAAGGAACTCATGACGGCG | GCCTTCTTCCAGGCTCCATT |
| *REV-ERBα* | TGCGTACTTCCCACCATCAC | CATGGCCACCTGTAGACTCC |
| *CLAUDIN-1* | TGCCTTGATGGTGATTGGCA | ATGACAGCCATCCGCATCTT |
| *OCCLUDIN* | CCGCCCATCACATCAGATCA | CAGCCGCCATGTACTCTTCA |
| *LGR5* | ATGGTGAAGCACATCGCTCT | CAGGAAGTGGGACGATCACC |
| *BMI1* | GGTCGAACTTGGTGTGTGTTC | TTTGCAGACTGGGGACAATGA |
| *AIF* | GACTGGAGCTGCTAAGCCAT | GGCAAACTGCTGTCCACAAG |
| *TPH1* | TTCGTCCTGTGGCTGGTTAC | CCAAAAGGGGAACGTGACCT |
| *TNFα* | ACCCTCACACTCAGATCATCTTCT | CAGATTGACCTCAGCGCTGAGTTG |
| *NF-Κβ* | CCTGAGTCTTTTGGACCGCT | GCAGGCTATTGCTCAACACG |
| *IL-6* | ACGATCCACTTCATCCTGCG | GGATGGTGTGTTCTGACCGT |
| *IL-10* | AGGGGCTTGCTCTTGCA | TGAAAACAAGAGCAAGGC |
| *GAPDH* | TGGTGAAGGTCGGAGTGAAC | ATGTAGTGGAGGTCAATGAATGG |

**Table S2** Significantly differentiated genera between the DF and NRF groups are shown, with *p* < 0.05 by Wilcoxon rank-sum test. The microbes present at more than 0.1% relative abundance were shown.

| Taxa  (Phylum) | Genus | DF  (Mean±SE) | NRF  (Mean±SE) | *P*-value |
| --- | --- | --- | --- | --- |
| Firmicutes | Ruminococcaceae_V9D2013_group | 1.44±1.54E-01 | 2.65±2.93E-01 | 0.00 |
|  | Ruminiclostridium_1 | 0.24±2.96E-02 | 0.43±4.65E-02 | 0.00 |
|  | [Eubacterium]_coprostanoligenes_group | 1.58±1.49E-01 | 0.94±8.29E-02 | 0.00 |
|  | uncultured_bacterium_f_Clostridiales_vadinBB60_group | 5.61±6.24E-01 | 8.64±5.95E-01 | 0.00 |
|  | uncultured_bacterium_f_Eubacteriaceae | 0.84±8.90E-02 | 1.34±1.34E-01 | 0.00 |
|  | [Eubacterium]_nodatum_group | 0.24±3.10E-02 | 0.12±1.70E-02 | 0.00 |
|  | uncultured_bacterium_f_Ruminococcaceae | 3.94±1.76E-01 | 3.34±1.72E-01 | 0.00 |
|  | Phascolarctobacterium | 1.45±1.67E-01 | 0.87±1.13E-01 | 0.00 |
|  | Subdoligranulum | 0.75±8.25E-02 | 0.49±5.77E-02 | 0.01 |
|  | Negativibacillus | 0.22±3.09E-02 | 0.14±2.86E-02 | 0.01 |
|  | uncultured_bacterium_f_Erysipelotrichaceae | 0.23±2.68E-02 | 0.33±3.37E-02 | 0.01 |
|  | Ruminiclostridium_6 | 1.64±1.48E-01 | 2.68±4.33E-01 | 0.02 |
|  | Papillibacter | 0.45±3.62E-02 | 0.57±3.56E-02 | 0.02 |
|  | Candidatus_Soleaferrea | 0.20±3.78E-02 | 0.12±2.39E-02 | 0.02 |
|  | uncultured_bacterium_f_Lachnospiraceae | 6.51±3.12E-01 | 7.65±4.98E-01 | 0.03 |
|  | Ruminococcaceae_UCG-010 | 3.09±1.61E-01 | 3.36±1.37E-01 | 0.03 |
|  | Ruminiclostridium_5 | 0.28±3.09E-02 | 0.36±3.86E-02 | 0.04 |
|  | Anaerovorax | 0.22±2.99E-02 | 0.16±2.32E-02 | 0.04 |
|  | Christensenellaceae_R-7_group | 1.96±2.31E-01 | 1.63±2.09E-01 | 0.05 |
| Bacteroidetes | Alistipes | 4.33±5.56E-01 | 1.44±2.20E-01 | 0.00 |
|  | Rikenella | 0.33±2.24E-01 | 1.75±5.23E-01 | 0.00 |
|  | uncultured_bacterium_f_Rikenellaceae | 1.42±3.76E-01 | 0.26±1.35E-01 | 0.00 |
|  | uncultured_bacterium_o_Bacteroidales | 0.15±6.64E-02 | 0.01±1.14E-02 | 0.01 |
|  | Butyricimonas | 0.24±4.42E-02 | 0.17±3.67E-02 | 0.04 |
|  | Rikenellaceae_RC9_gut_group | 0.00±0.00E+00 | 0.34±3.14E-01 | 0.04 |
| Proteobacteria | uncultured_bacterium_o_Rhodospirillales | 0.74±1.19E-01 | 0.29±4.84E-02 | 0.00 |
|  | Desulfovibrio | 1.91±1.32E-01 | 1.58±9.47E-02 | 0.03 |
|  | Escherichia-Shigella | 0.71±7.07E-01 | 0.0±3.60E-03 | 0.04 |
| Patescibacteria | Candidatus_Saccharimonas | 0.33±6.01E-02 | 0.19±2.56E-02 | 0.03 |
| Tenericutes | uncultured_bacterium_o_Mollicutes_RF39 | 1.80±1.24E-01 | 1.43±1.12E-01 | 0.01 |
| Synergistetes | Synergistes | 0.41±1.36E-01 | 0.06±3.11E-02 | 0.01 |
| Epsilonbacteraeota | Campylobacter | 0.18±6.60E-02 | 0.24±3.52E-02 | 0.00 |

**Table S3** Rhythmic analysis of microbes at genus level.

| Feeding pattern | Taxa  (Phylum) | Genus | Adj. P | Phase | Ampl | Period |
| --- | --- | --- | --- | --- | --- | --- |
| DF | Firmicutes | Intestinimonas | 1.49E-02 | 2 | 9.00E-02 | 24h |
|  |  | uncultured_bacterium_f_Clostridiales_vadinBB60_group | 2.63E-02 | 20 | 2.01E+00 | 24h |
|  |  | uncultured_bacterium_f_Atopobiaceae | 3.31E-02 | 14 | 5.48E-02 | 24h |
|  |  | Flavonifractor | 4.41E-02 | 20 | 8.58E-02 | 24h |
|  | Cyanobacteria | uncultured_bacterium_o_Gastranaerophilales | 4.21E-02 | 20 | 9.89E-02 | 24h |
| NRF | Firmicutes | Lachnospiraceae_NK4A136_group | 1.43E-03 | 8 | 7.17E-01 | 24h |
|  |  | Roseburia | 4.04E-03 | 8 | 4.05E-02 | 24h |
|  |  | Ruminococcaceae_UCG-013 | 2.76E-02 | 10 | 1.88E-01 | 24h |
|  |  | [Eubacterium]_xylanophilum_group | 4.12E-02 | 10 | 4.77E-02 | 24h |

**Reference**

Guo, Y., Wang, Q.J., Zhang, K.H., Yao, C.Y., Huang, J., Li, Q., et al. (2020). Night‐restricted feeding improves locomotor activity rhythm and modulates nutrient utilization to accelerate growth in rabbits. *FASEB J*. 2020;35:e21166. doi: 10.1096/fj.202001265RR.
